# Heart Rate Variability and Clinical Features as Predictors of Atrial Fibrillation Recurrence After Catheter Ablation: A Pilot Study

**DOI:** 10.3389/fphys.2021.672896

**Published:** 2021-05-25

**Authors:** Javier Saiz-Vivo, Valentina D. A. Corino, Robert Hatala, Mirko de Melis, Luca T. Mainardi

**Affiliations:** ^1^Medtronic Bakken Research Center B.V., Maastricht, Netherlands; ^2^Department of Electronics, Information and Bioengineering, Politecnico di Milano, Milan, Italy; ^3^Department of Arrhythmias and Cardiac Pacing, National Institute of Cardiovascular Diseases, Bratislava, Slovakia

**Keywords:** atrial fibrillation, classification, ablation, implantable cardiac monitor (ICM), heart rate variability, recurrences, machine learning

## Abstract

Single-procedure catheter ablation success rate is as low as 52% in atrial fibrillation (AF) patients. This study evaluated the feasibility of using clinical data and heart rate variability (HRV) features extracted from an implantable cardiac monitor (ICM) to predict recurrences in patients prior to undergoing catheter ablation for AF. HRV-derived features were extracted from the 500 beats preceding the AF onset and from the first 2 min of the last AF episode recorded by an ICM of 74 patients (67% male; 57 ± 12 years; 26% non-paroxysmal AF; 57% AF recurrence) before undergoing their first AF catheter ablation. Two types of classification algorithm were studied to predict AF recurrence: single classifiers including support vector machines, classification and regression trees, and K-nearest neighbor classifiers as well as ensemble classifiers. The sequential forward floating search algorithm was used to select the optimum feature set for each classification method. The optimum weighted voting method, which used an optimum combination of the single classifiers, was the best overall classifier (accuracy = 0.82, sensitivity = 0.76, and specificity = 0.87). Clinical and HRV features can be used to predict rhythm outcome using an ensemble classifier which would enable a more effective pre-ablation patient triage that could reduce the economic and personal burden of the procedure by increasing the success rate of first catheter ablation.

## Introduction

Catheter ablation, specifically pulmonary vein isolation (PVI), has become a common treatment over the decades for atrial fibrillation (AF) patients, especially those highly symptomatic (Arbelo et al., 2014) or those where antiarrhythmic drug therapy has not been sufficient (or tolerated) for rhythm stabilization (Kirchhof et al., 2017). However, the long-term outcomes of catheter ablation in AF reported single-procedure success rates as low as 66.6% in paroxysmal AF (PAF) patients and 51.9% in non-paroxysmal AF (NPAF) patients (Ganesan et al., 2013). Several well-established scoring systems aimed at predicting rhythm outcome after catheter ablation, including thromboembolic risk predictors like CHADS_2_ or CHA_2_DS_2_-VASc, have shown modest prediction capabilities (Letsas et al., 2014). Other specific rhythm outcome predictors such as APPLE (Kornej et al., 2015), SUCCESS (Jud et al., 2019), and MB-LATER (Mujović et al., 2017) have achieved better results. However, most studies done so far have the drawback of relying on 24-h Holter monitoring to detect AF recurrences, which was shown to have a rather poor detection rate for subclinical AF of 5.5% (Ramkumar et al., 2018).

Implantable cardiac monitors (ICMs) offer the advantage of long-term monitoring and use highly sensitive AF detection algorithms, with detection rates of up to 96% (Hindricks et al., 2010). These devices continuously classify the heart rhythm of a patient by analyzing its cardiac cycle and, in addition, store the R–R intervals of the beats preceding the AF episode and the first beats of the AF episode. Hence, heart rate variability (HRV)-derived features can be extracted. HRV has been proven to be a predictor of chronic AF recurrence after electrical cardioversion (Lombardi et al., 2001; Akyürek et al., 2003), and extensive work has been done in describing the changes in HRV features before and after ablation (Kocovic et al., 1993; Hsieh et al., 1999; Kang et al., 2014; Seaborn et al., 2014).

This article proposes the use of common HRV-derived features in conjunction with clinical data to predict recurrences within the first 12 months after catheter ablation in a continuously monitored patient population. To accomplish this, the article will evaluate several single classification methods including support vector machines (SVM), with linear (Christianini and Shawe-Taylor, 2013), polynomial (SVMp), and Gaussian (SVMg) kernels (Djerioui et al., 2019), classification and regression trees (CART), and K-nearest neighbor (KNN) algorithms. In addition, the capabilities of ensemble learning methods (Hosseini et al., 2020) in which a weighted combination of the single classifiers is used as the predictor of AF recurrence will be explored.

## Materials and Methods

### Patient Population

This retrospective study included patients from two cohorts: the Reveal LINQ usability study (ClinicalTrials.gov identifier: NCT01965899), a multicenter single-arm clinical study, and clinical data derived from a single center with extensive experience in evaluating the long-term outcome of AF ablation by means of ICM (Bou Ezzeddine et al., 2015). The patients of both cohorts provided written informed consent, and the study protocols were reviewed and approved by the Human Research Ethics Committee of each participating institution.

**FIGURE 1:**
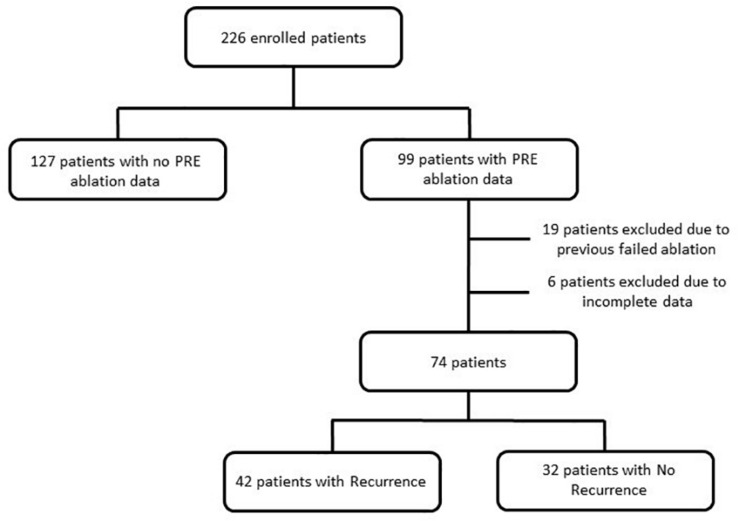
Flow diagram describing the source population.

Out of the 226 enrolled patients, only 99 had pre-ablation data, out of which 19 were excluded due to previously failed ablation and another six due to incomplete data such as no medical and/or ablation records. A schematic presentation describing the patient cohort can be found in Figure 1. The selected 74 patients (67% male; 57 ± 12 years; 26% NPAF) were divided into two classes: those with AF recurrence (*R* = 42 patients, 57% of the total) and those with no AF recurrence (NR = 32, 43%). AF recurrence was defined as presence of an AF episode longer than 2 min as recorded by the ICM after a 3-month blanking period following catheter ablation. The blanking period of 3 months is used as suggested by the 2012 Consensus Statement of Catheter and Surgical Ablation of Atrial Fibrillation to report the efficacy of the ablation as early recurrences could be caused by post-ablation inflammation or short-term autonomic imbalance (Calkins et al., 2012). Both cohorts had more than 12 months follow-up for AF recurrence after catheter ablation.

**FIGURE 2:**
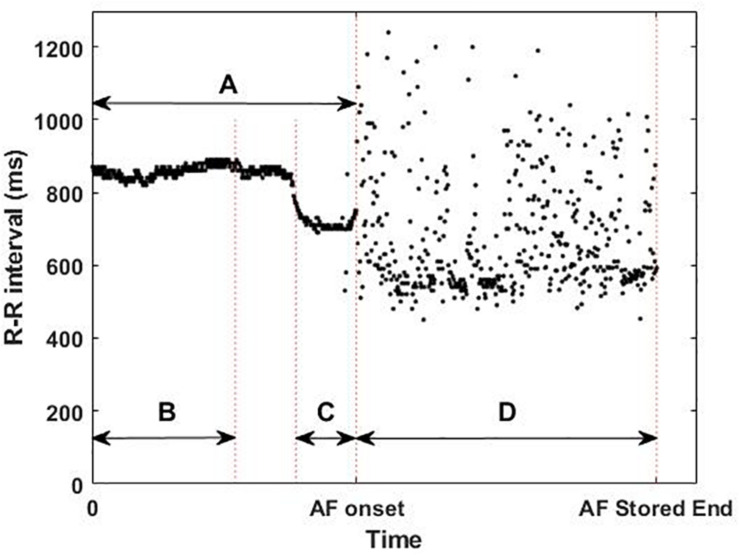
Example of available implantable cardiac monitor data: **(A)** whole flashback, **(B)** first 300 beats of the flashback, **(C)** last 100 beats of the flashback, and **(D)** first 2 min of the last atrial fibrillation (AF) episode recorded before catheter ablation, illustrating the short-term changes in the R–R intervals (ms) prior to the AF onset and the differences in R–R intervals between the flashback **(A)** and the AF episode **(D)**.

The ICM was implanted 5.9 ± 3.8 months before the ablation procedures, which were classified as PVI or PVI plus Extra Lesions. These long monitoring periods ensure the detection of the AF onset of both paroxysmal and non-paroxysmal patients. The devices used in the usability and the single-center studies were Reveal LINQ and Reveal XT (Medtronic Inc, Minneapolis, MN, United States), respectively, and were implanted within the fourth intercostal space (V2–V3 electrode orientation). Both devices sense and detect the rhythm with a sampling frequency of 256 Hz and then, to optimize memory slots, store the R-peak timestamps and the ECG of the first 2 min of the AF episodes. For the last AF episode recorded, the device also stores the timestamps of the beats preceding the AF onset (Flashback). For the analysis, the first beats of the last recorded AF episode (477 ± 71 beats) before the catheter ablation and its Flashback (483 ± 33 beats) were extracted (D and A in Figure 2). The last AF episodes occurred between 1 and 183 days before the ablation.

### Data Collection and Feature Extraction

From these two types of signals (Flashback and last AF episode) 4 different areas of interest (AoI) were defined: the whole Flashback (A), the first 300 beats of the Flashback (B), the last 100 beats of the Flashback (C), and the first beats of the AF episode (D) as shown in Figure 2.

Classical HRV-derived features describing the variability and irregularity of the R–R intervals (see Table 1) were computed from the different AoIs and then categorized into four groups: whole Flashback (FB, AoI: A), last 100 beats of Flashback (L100, AoI: C), and delta and first beats of AF episode (AF, AoI: D). Delta was defined as the percentage difference between the first 300 beats of the Flashback (AoI: B) and the last 100 beats of the Flashback (AoI: C) and was computed as:

Delta⁢(feature)=First⁢300⁢(feature)-Last⁢100⁢(feature)First⁢ 300⁢(feature)*100

**TABLE 1:** Description of the heart rate variability features used in the analysis.

Features	Short descriptions	References
Mean	Mean of the R–R intervals (ms)	Camm et al., 1996
pNN50, pNN20	Percentage of the interval differences of successive R–R intervals greater than 50 and 20 ms (%)	Mietus et al., 2002
RMSSD	Mean square differences of successive R–R intervals (ms)	Camm et al., 1996
SDNN	Standard deviation of the R–R intervals (ms)	Camm et al., 1996
TINN	Triangular interpolation of interval histogram (ms)	Camm et al., 1996
TRI	Triangular index: integral of the density distribution	Camm et al., 1996
ApEn, SampEn	Approximate and sample entropy: estimators of the complexity of the R–R series	Delgado-Bonal and Marshak, 2019
SD1, SD2, SD1SD2ratio	Geometric descriptors of the Poincare plot	Tayel and AlSaba, 2015
DFA α1, α2	Scaling exponents of short- and long-term fluctuations of the R–R intervals	Peng et al., 1995

with the aim of studying the changes occurring within the Flashback itself.

In addition to variability and irregularity features, clinical information such as the age, AF type (paroxysmal or non-paroxysmal), hypertension presence, and ablation type (PVI or PVI plus extra lesions) were included in the analysis. A total of 14 R–R interval variability and irregularity features were computed for the four areas of interest, except pNN50 and pNN20 for Delta as the values for the first 300 beats of these features were 0 for some patients, and the relative change could not be computed. A total of 58 features were considered per patient, including the four clinical features and the variability and irregularity features.

### Classification

**FIGURE 3:**
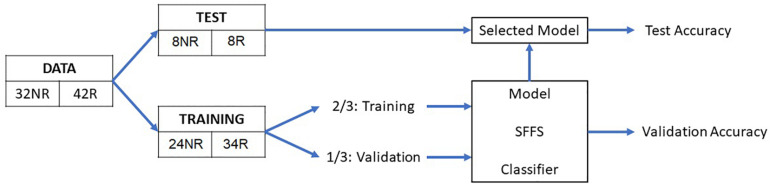
Schematic representation of the overall method. NR, no recurrence; R, recurrence; SFFS, sequential forward floating search.

Based on the extracted features, eight classification algorithms were evaluated to predict AF recurrence after a 3-month blanking period. As a first step, a test set was randomly selected, containing 22% of the data (eight patients with recurrence and eight with no recurrence), which was used to evaluate the performance of the classifiers on never-before-seen data. The remaining 78% of the observations were processed by the classification algorithm, using 2/3 of the data to train the classification model (the training set) and 1/3 to validate the trained model (validation set) as illustrated in Figure 3. The classification algorithm included the feature selection and the model training, out of which the validation performance metrics were computed. As a feature selection tool, the sequential forward floating search (SFFS) algorithm was used. This algorithm considers and analyzes subsets with different number of features by iteratively selecting the features that increase the overall accuracy of the model (Somol et al., 2006). Further information on the algorithm can be found in the Supplementary Material.

The trained model was evaluated on the test set, on which the performance metrics were computed.

The classification was performed using two types of classifiers: single classifiers and ensemble classifiers. The single classifiers were SVM, CART, and KNN; the ensemble classifiers were mean voting (MV), accuracy weighted voting (AWV), and optimum weighted voting (OWV).

For the ensemble classifiers, the five classifications of the single classifiers were weighed and combined to classify a given observation *x*: *C*(*x*), which is the result of the scalar product between the weight vector (**W**) and the voting vector (**V**(*x*)):

$$C\,\left(x\right)=\left\{ \begin{array}{c} 1\,i\,f\,\text{W}\cdot \text{V}\,{\left(x\right)}^{T}>0.5\\ 0\,i\,f\,\text{W}\cdot \text{V}\,{\left(x\right)}^{T}\leq 0.5 \end{array}\right.$$

where, the weight vector:

$$\text{W}=\left[W_{\text{SVM}},W_{\text{SVMp}},W_{\text{SVMg}},W_{\text{CART}},W_{\text{KNN}}\right]$$

contains the weights for the single classifiers (*W*_*i*_ ∈ [0,1],∑*W*_*i*_=1), and the voting vector:

$$\text{V}\,\left(x\right)=\left[V_{\text{SVM}}\,\left(x\right),V_{\text{SVMp}}\,\left(x\right),V_{\text{SVMg}}\,\left(x\right),V_{\text{CART}}\,\left(x\right),V_{\text{KNN}}\,\left(x\right)\right]$$

is the binary classification of the observation *x* made by the single classifiers.

Each ensemble algorithm had a different weight configuration. The MV algorithm was defined as the average of all the classifications; thus, the single classifiers have the same weight:

$$W_{\text{SVM}}=W_{\text{SVMp}}=W_{\text{SVMg}}=W_{\text{CART}}=W_{\text{KNN}}=0.2.$$

In the AWV method, the weights are proportional to the accuracy on the validation set of the single classifiers, thus being:

Wi=Accuracyi∑Accuracyi⁢with⁢i=SVM′′,′SVMp′,′SVMg′,′CART′,′KNN′

Finally, for the OWV method, all possible weight combinations (considering a step of 0.1 for each weight) were iteratively evaluated, and the set of weights that maximizes the overall accuracy of the validation set was selected.

Leave-*p*-out cross-validation (where *p* is 1/3 of the data) was performed with 100 bootstrap repetitions, i.e., all the above-mentioned steps were repeated 100 times, randomizing the patient selection and allowing both patients with AF recurrence and those without to be part of the training and validation phases. Performance metrics such as accuracy, sensitivity, and specificity were then averaged over the repetitions, while F1-score was computed from the averaged confusion matrix, and were computed as:

$$\text{Accuracy}=\frac{\text{TP}+\text{TN}}{\text{TP}+\text{TN}+\text{FP}+\text{FN}}$$

$$\text{Sensitivity}=\frac{\text{TP}}{\text{TP}+\text{FN}};\mathrm{Specificity}=\frac{\text{TN}}{\text{TN}+\text{FP}}$$

$$\mathrm{F1}-\text{score}=2\cdot \frac{\text{PPV}\cdot \text{Sensitivity}}{\text{PPV}+\text{Sensitivity}}=\frac{2\,T\,P}{2\,T\,P+\text{FP}+\text{FN}}$$

where TP is true positive, TN is true negative, FP is false positive, FN is false negative, and PPV is positive predictive value. The feature extraction, selection, and classification were conducted using Matlab R2019b (The Mathworks Inc., Natick, MA, United States).

### Statistical Analysis

The statistical properties of the optimum set of features used by the classification method with the highest accuracy were analyzed. Continuous data are presented as mean ± SD if the null hypothesis *H_0* of the Shapiro–Wilk test (*H_0*: data is normally distributed) was not rejected, and these were compared with the unpaired Student’s *t*-test. Otherwise, continuous data are presented as median (IQR), with IQR being the interquartile range, and compared using the Mann–Whitney *U* test. Conversely, categorical data is presented as absolute frequency (relative frequency in percentage) and compared with the Pearson chi-square method. A *p*-value < 0.05 was considered for the rejection of the null hypothesis and set as the level of significance. All statistical analyses were conducted using SPSS, version 23 (SPSS Inc., Chicago, IL, Illinois).

## Results

The available clinical baseline characteristics of the 74 patients are shown in Table 2. Even though there are no statistically significant differences between the clinical baseline characteristics used in the analysis of the patients with and without recurrences (age, paroxysmal AF, hypertension, and extra lesions), patients with AF recurrence were, on average, 3.6 years older than those without. There was also a higher proportion of paroxysmal AF patients and a lower proportion of arterial hypertension patients among those who did not have AF recurrences. Diabetes, coronary artery disease, and stroke were excluded from the analysis as these features were heavily underrepresented. The HRV-derived features for each group of interest are shown in Supplementary Table 1 as mean ± standard deviation for normally distributed data and as median (IQR) for non-normally distributed data. Only pNN20 for the whole Flashback, delta triangular index, and sample entropy in the AF episode have a statistically significant difference between patients with and without AF recurrences.

**TABLE 2:** Clinical baseline characteristics of the patients that had no recurrence and those who did.

Feature	No recurrence (NR = 32, 43%)	Recurrence (*R* = 42, 57%)	*p*-value
Age, years	55.47 ± 12.79	59.12 ± 11.55	0.20
Paroxysmal atrial fibrillation	26 (81.3%)	29 (69.1%)	0.18
Hypertension	19 (59.4%)	26 (61.9%)	0.83
Diabetes	1 (3.1%)	5 (11.9%)	<0.001
Coronary artery disease	0	5 (11.9%)	<0.001
Stroke	3 (9.4%)	2 (4.8%)	<0.001
Monitoring time pre-ablation, months	8.1 ± 3.7	4.3 ± 2.9	<0.001
Extra lesions	5 (15.6%)	10 (23.8%)	0.39

**FIGURE 4:**
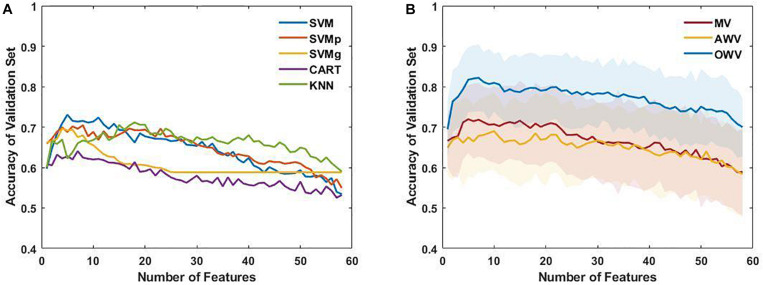
**(A)** Mean accuracy of the single classifiers on the validation set, plotted as a function of the number of selected features. **(B)** Mean and standard deviation of the accuracy of the multiple classifiers (line and colored area, respectively) for each subset of features on the validation set. AWV, accuracy weighted voting; CART, classification and regression trees; KNN, K-nearest neighbors; OWV, optimum weighted voting; SVM, support vector machine; SVMg, support vector machine Gaussian kernel; SVMp, support vector machine polynomial kernel.

**FIGURE 5:**
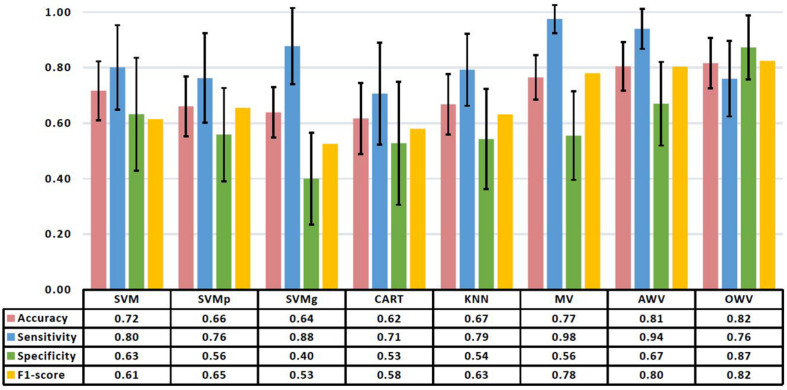
Mean and standard deviation of the performance metrics on the test set of the different classification methods. The table shows the mean values. AWV, accuracy weighted voting; CART, classification and regression trees; KNN, K-nearest neighbors; OWV, optimum weighted voting; SVM, support vector machine; SVMg, support vector machine Gaussian kernel; SVMp, support vector machine polynomial kernel.

Firstly, the single classifiers were evaluated by computing the accuracy on the validation set for subsets with an increasing number of features as selected by the SFFS. Figure 4A shows the mean accuracies on the validation set of the single classifiers as a function of the number of selected features. It can be observed that all the classifiers reach a maximum of accuracy for the validation set with a subset containing less than 10 features except KNN (number of features = 18). For each single classifier, the feature subset that maximizes the validation accuracy is selected as the optimum feature set, which is used to evaluate the test using the trained model The performance evaluators for the test set were then computed in every iteration, and Figure 5 depicts the mean and standard deviation. The F1-score, however, was computed from the averaged confusion matrix (also shown in Figure 5), as only the average score was of interest to compare different classifiers

When working with never-seen data, SVM had the highest accuracy (0.72 ± 0.11) and specificity (0.63 ± 0.20), while SVMg had the highest sensitivity (0.88 ± 0.14). The highest F1-score (0.65) was obtained by SVMp.

The single classifiers were then combined in an ensemble classifier in which a weighted combination of the single classification is used to compute the final classification. Figure 4B shows the mean (bold line) and the standard deviation (shaded area) of the accuracy of the validation set for the different ensemble classifiers. Similar to the single classifiers, the maximum accuracy was reached with less than 10 features. The optimum feature set was determined as the subset that maximized the accuracy on the validation set, and the results for the ensemble classifiers’ comparison are shown in Figure 5.

The best overall classifier with the highest F1-score (0.82) is the OWV method, in which the weights used to combine the different single classifiers are evaluated in each iteration. This method also has the highest accuracy (0.82 ± 0.09) and specificity (0.87 ± 0.12) on the test set, while MV has the highest sensitivity (0.98 ± 0.05).

This OWV method of classification used a set of seven features combining geometric delta features (“delta SD1SD2ratio”) with complexity delta features (“delta ApEn”), statistical delta features (“delta RMSSD”), geometric AF features (“SD2 AF”), statistical AF features (“pNN20 AF”), statistical Flashback features (“pNN20”), and clinical features (“extra lesions”).

**FIGURE 6:**
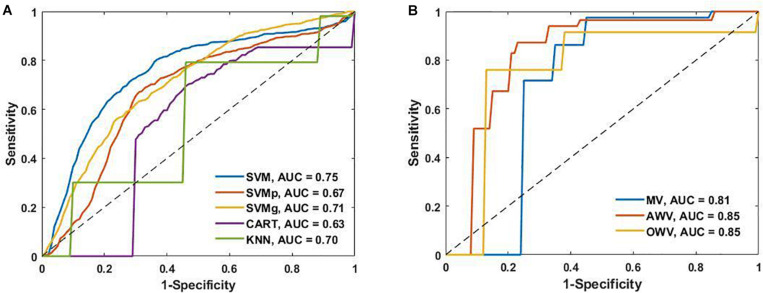
**(A)** Receiver operating characteristic (ROC) curves of the single classifiers and area under the curve (AUC). **(B)** ROC curves of the ensemble classifiers and the AUC. AWV, accuracy weighted voting; CART, classification and regression trees; KNN, K-nearest neighbors; OWV, optimum weighted voting; SVM, support vector machine; SVMg, support vector machine Gaussian kernel; SVMp, support vector machine polynomial kernel.

To provide added information and insight on the performance of the different classification models, the receiver operating characteristic curves from the single and the ensemble classifiers are shown in Figure 6 alongside their area under the curve (AUC) values. SVM had the highest AUC value (0.75) of the single classifiers, while AWV and OWV both obtained the highest overall AUC value (0.85).

**FIGURE 7:**
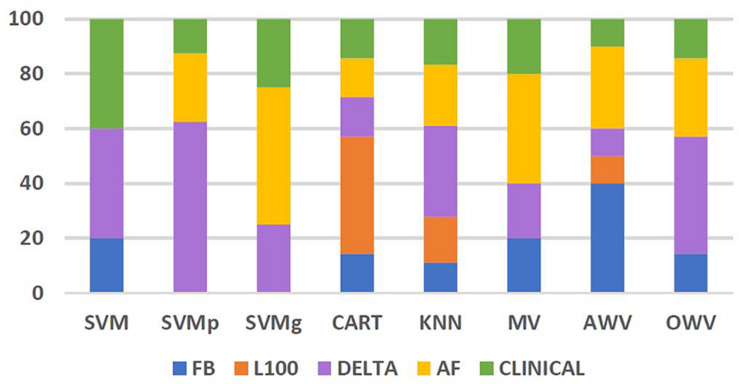
Percentage of features from each feature group used by the different classification methods. AF, atrial fibrillation; AWV, accuracy weighted voting; CART, classification and regression trees; FB, flashback; KNN: K-nearest neighbors; L100, last 100 beats; OWV, optimum weighted voting; SVM, support vector machine; SVMg, support vector machine Gaussian kernel; SVMp, support vector machine polynomial kernel.

The frequency of use of each of the feature groups, i.e., FB: Flashback, L100: Last 100, Delta, AF, and clinical by the different classification methods, was also analyzed. Figure 7 shows the percentage of each feature group used by the different methods. Features from feature group Delta were the most frequently used by the different classifiers. On average, the classifiers used features from Delta group for 31% of their selected features, reaching 63% of the selected features for the SVMp method. However, it is worth noting that every classifier took at least “extra lesions” as one of the optimum features in their feature list, and classifiers such as SVM and SVMg had features from the clinical group comprising more than 25% of their features, with SVMg being the classifier with the highest percentage of use (40%).

## Discussion

The main finding of this study is that a reduced set of HRV and clinical features extracted from an ICM can be used by an ensemble classifier to predict AF recurrence with a mean accuracy higher than 0.8 in patients that underwent single-procedure catheter ablation. If confirmed by future studies, these findings are potentially of significant clinical relevance for several reasons: first, catheter ablation of the AF substrate is a procedure with high economic and personal burden; secondly, due to the epidemic character of AF prevalence, these interventions cannot be offered (even in countries with developed healthcare systems) to all patients; and third, the selection of patients with higher probability of long-term elimination of AF has high priority. Until now, the various clinical scoring systems based on phenotypic biomarkers used to this aim generate a rather poor prediction of limited clinical usefulness.

HRV has been extensively studied with respect to procedural outcome by analyzing the changes in HRV features before and after ablation (Kocovic et al., 1993; Hsieh et al., 1999; Kang et al., 2014; Seaborn et al., 2014). However, these studies mainly describe the effect of ablation on HRV and use non-continuous Holter monitoring of the patients. To the best of our knowledge, this is the first study that reports the combination of classical HRV and clinical features to predict AF recurrence in a continuously monitored ablation cohort using a variety of classification methods.

Although all of the different HRV and clinical features were initially introduced in the algorithm, SFFS iteratively selected the optimum set, and only one of the eight different methods used had a peak performance with more than 10 features and OWV only used seven: delta SD1SD2 ratio, delta ApEn, delta RMSSD, pNN20 AF, SD2 AF, and extra lesions. Out of the seven features found as optimum, only one was a clinical feature, while the others were classical HRV features extracted from three different feature groups: delta, Flashback, and AF. RMSSD and Poincare descriptor SD1SD2 ratio both describe the variability of the R–R intervals, and so when analyzing the correlation between the features, it was not surprising to observe that they had an *r* = 0.506, *p* < 0.01, in the two-tailed Pearson correlation test. The rest of the features had a weak correlation between them.

The most used feature group appeared to be delta, with an average use of 31% in the different classifiers and a maximum of 63% in SVMp, which shows the importance of studying the onset of the AF contained within the Flashback.

Several studies have investigated different methods of AF recurrence prediction by analyzing the impact of different clinical scores. While thromboembolic risk predictors like CHADS_2_ or CHA_2_DS_2_-VASc showed a relatively modest prediction (Letsas et al., 2014), other specific rhythm outcome predictors such as APPLE (Kornej et al., 2015), SUCCESS (Jud et al., 2019), and MB-LATER (Mujović et al., 2017) have been introduced as yielding better results. The APPLE score [one point for age > 65 years, persistent AF, impaired eGFR < 60 ml/min/1.73 m^2^, left atrial (LA) diameter ≥ 43 mm, and ejection fraction (EF) < 50%] was originally developed to predict AF recurrences after first ablation, with area under the receiver operating characteristic curve (AUC) of 0.634 (Kornej et al., 2015), but it has also been tested to predict recurrence in repeated ablations, with AUC of 0.617 (Kornej et al., 2017). Based on this score, the SUCCESS score was created by adding one point for each preciously performed ablation, and although it did demonstrate an improvement over APPLE (AUC 0.657 *vs* 0.620), the findings were not significant in the study (Jud et al., 2019). The MB-LATER score [male, bundle branch block, left atrium ≥ 47 mm, type of AF (paroxysmal, persistent, or long-standing persistent), and ER - AF = early recurrent AF] was associated with patients who will develop very late AF recurrence, i.e. recurrence documented more than 12 months after the ablation procedure (AUC 0.782) (Mujović et al., 2017), and was also proven to predict late AF recurrence (AUC 0.62) (Potpara et al., 2019). However, this score has a drawback in that it uses early recurrence of AF as a feature, and it cannot be used as a baseline predictor. All these scoring systems have the disadvantage of relying on conventional Holter devices to detect AF recurrence in patients and the need of image-based parameters such as LA diameter or EF, while the proposed method uses easily obtainable clinical information and classical HRV features extracted from an ICM which continuously monitors the patient.

In the review of AF recurrence predictors developed by Balk et al. (2010) the relationship between success of ablation for AF and clinical features was systematically evaluated. The multivariable analyses showed that neither age, AF type, nor hypertension showed a significant association to ablation success. However, in the case of age, Balk et al. (2010) suggested that this result was due to the limitations of the existing literature rather than a true lack of association, as only relatively young patients were included in the analyses (40–70 years). The patient population analyzed in this study was relatively young (57.5 ± 12.15 years old), and this could be the reason why age was not included in the final feature set. Even though the multivariate analyses performed by Balk et al. (2010) failed to show a significant association between AF type and ablation success, the univariate analysis found that patients with NPAF had a 60% increased risk of AF recurrence compared to those with PAF, and it was hypothesized that it would be a good clinical indicator of the likelihood of AF recurrence. In this study, AF type was chosen by only two of the eight classification methods, and while one was AWV, which had the second highest F1 score, the other one was SVMp, which had one of the lowest scores. This could be explained by the under-representation in the patient population of non-paroxysmal patients (25.7%) which translated into 18.8% of those which did not have AF recurrence and 31.0% of those who did. Ablation type was the only feature used by every method: from these preliminary results, patients that had extra lesions also had a higher chance of having AF recurrence. Although there are some confounding factors to consider, i.e., the need of extra lesions may be due to a more advanced AF with a higher presence of fibrotic tissue in the atria; the extra scar tissue could be the foci of new re-entry circuits that could develop and sustain AF. Nonetheless, this feature was also heavily biased, as most of the patients underwent PVI ablations and only 15 patients (20.3%) also had extra lesions, so further work would have to confirm this.

This retrospective study was made using a limited patient population from two different cohorts with different clinical information, which limited the clinical features that could be used to those which were collected in both studies. Furthermore, the features that were included, such as AF type and ablation type, were heavily biased, as persistent patients (26% of the total number of patients) and ablation strategies with extra lesions (20%) were underrepresented. However, the main part of this study was focused on HRV features, and even though the use of the clinical features increased the accuracy and future work should be done to understand their impact, the presented classification method and the results are still clinically relevant. The data were extracted from the Reveal LINQ ICM which automatically detected AF episodes longer than 2 min and, due to memory restrictions, stored the R–R intervals of the episodes detected and their Flashbacks. Therefore, episodes longer than 30 s, which are defined as AF episodes by the guidelines, but shorter than 2 min were undetected by the ICM. The lack of stored ECG signals also limited the number of features that could be extracted and restricted the analysis to HRV-derived features that could be extracted from the R–R intervals. Despite these drawbacks, the advantage of having continuous monitoring of the patients before and after the ablation greatly outweighs the disadvantages of possible information loss due to device resolution or memory restrictions. Although the study shows promising results and serves as proof of the feasibility of the method described, being a pilot study, the results should be validated on an external database.

Recurrence of AF after ablation can be predicted with varying degrees of accuracy using simple classification methods and an iteratively selected feature set of easily obtainable HRV and clinical features. The best approach is an optimally weighted combination of single classifiers which uses HRV (Poincare descriptors SD1SD2 ratio, pNN20 approximate entropy, RMSSD, and triangular index) and clinical (extra PVI lesions) features. This could be a first step into a more effective pre-ablation patient triage that could reduce the economic and personal burden of the procedure by increasing the success rate of the first catheter ablation to achieve long-term AF termination.

## Data Availability Statement

The data analyzed in this study is subject to the following licenses/restrictions. Dataset owned by Medtronic and by the Department of Arrhythmias and Cardiac Pacing, National Institute of Cardiovascular Diseases of Slovakia. Requests to access these datasets should be directed to JS-V and RH.

## Author Contributions

JS-V contributed to the design of the study, wrote and implemented the code, analyzed the results, created the figures and tables, and wrote the first draft of the manuscript. RH designed the clinical study and obtained and provided clinical data. MM provided clinical data, contributed to the design of the study, analyzed the results, and supervised the project. VC wrote code, contributed to the design of the study, analyzed the results, and supervised the project. LM contributed to the design of the study, analyzed the results, and supervised the project. All authors contributed to manuscript revision and read and approved the submitted version.

## Conflict of Interest

MM is a Medtronic employee and JS-V is affiliated to Medtronic and supported by an EU grant. The remaining authors declare that the research was conducted in the absence of any commercial or financial relationships that could be construed as a potential conflict of interest.
