# Supplementary material for: Heart Rate Variability and Clinical Features as Predictors of Atrial Fibrillation Recurrence After Catheter Ablation: A Pilot Study
Source: Front Physiol. 2021 May 25;12:672896. doi: 10.3389/fphys.2021.672896 (PMC8185295; doi:10.3389/fphys.2021.672896)
Supplement: Supplementary file 1 [file Data_Sheet_1.docx]

**SUPPLEMENTARY MATERIAL**

Briefly, starting from an empty set of features ($S_{k}$), the feature $f_{i}$ that maximizes the objective function (accuracy) when combined with $S_{k}$, is added. After the forward step is repeated (and a minimum of 3 features are already added) SFFS performs backward steps in which the feature that makes the objective function increase when removed from $S_{k}$, is removed. A schematic representation of the algorithm is shown in Figure S1


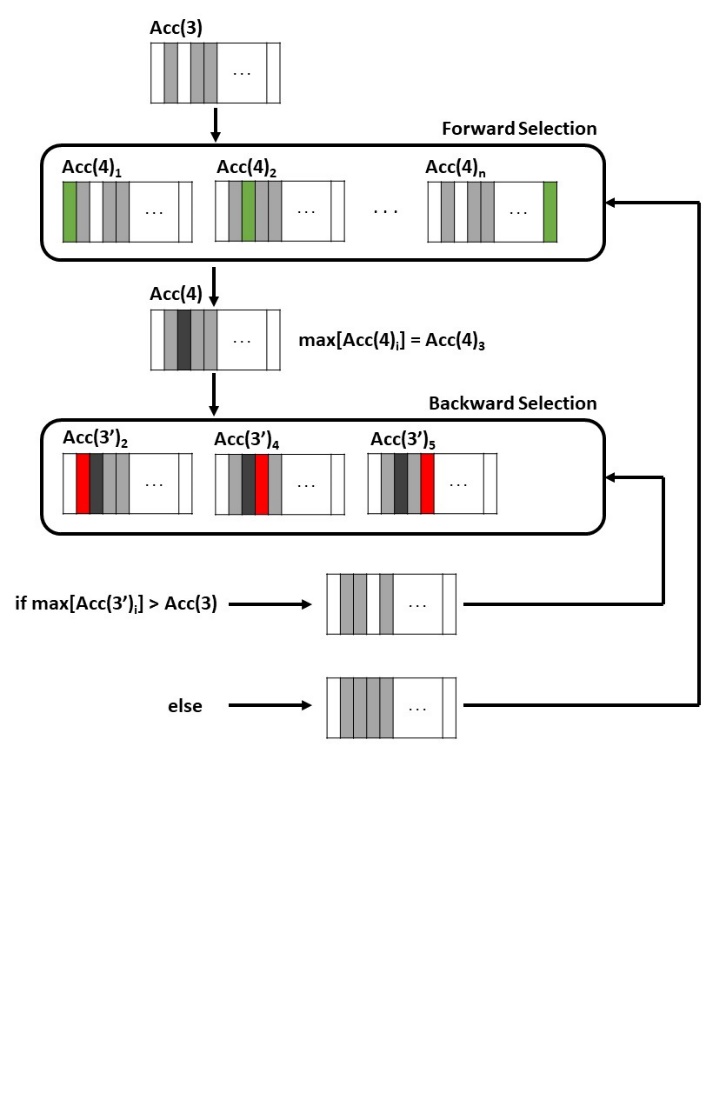


Figure *S1*: Schematic representation of the SFFS algorithm, as an example starting with three features already selected. The top rectangle represents the current set of 3 chosen features (grey lines) along with the unchosen features (white lines) which have an accuracy Acc(3). In the Forward Selection step, each of the remaining features are iteratively added (green line) and the new accuracy Acc(4)_i_ computed. Once the feature producing the maximum accuracy is selected and included in the selected set (black line), the Backward Selection step takes place. Each of the selected features, except the newly selected one, are removed from the selected feature set (red line) and the new accuracy Acc(3’)_i_ computed. If the maximum Acc(3’)_i_ is higher than the previous Acc(3), the feature is removed from the selected features set and the Backward Selection is repeated, otherwise the algorithm goes to Forward selection where Acc(5)_i_ are evaluated. Modified from (Corino et al. 2018).

Table S1: Parameters computed for each Feature, Group and Class. Continuous normally distributed parameters are shown as mean ± standard deviation while continuous non-normally distributed parameters are shown as median (interquartile range). Pair of parameters with a p-value < 0.05 (*).

| **Feature** | **FB NR** | **FB R** | **L100 NR** | **L100 R** | **Delta NR** | **Delta R** | **AF NR** | **AF R** |
| --- | --- | --- | --- | --- | --- | --- | --- | --- |
| Mean (ms) | 824.99 ± 173.50 | 826.48 ± 172.54 | 816.73 ± 176.47 | 820.89 ± 185.46 | 0.79 (16.52) | 0.48 (11.12) | 679.73 (264.98) | 741.27 (290.21) |
| pNN50 (%) | 13.63 (35.87) | 39.75 (66.66) | 19.19 (43.94) | 38.72 (61.62) | - | - | 43.74 (27.57) | 55.11 (31.07) |
| pNN20 (%) | **51.36 (45.69)*** | **65.08 (48.50)*** | 57.07 (48.48) | 64.86 (47.47) | - | - | 70.28 ± 13.24 | 75.66 ± 13.31 |
| RMSSD (ms) | 96.29 (75.46) | 129.83 (146.31) | 93.07 (93.03) | 122.87 (126.12) | -15.01 (102.05) | -19.81 (49.18) | 172.95 (69.66) | 205.56 (124.71) |
| SDNN (ms) | 103.64 (81.71) | 128.97 (82.34) | 89.48 (77.54) | 112.06 (63.52) | -9.10 (87.78) | -4.62 (35.87) | 138.28 (107.20) | 167.95 (96.26) |
| TINN (ms) | 0.14 (0.10) | 0.17 (0.14) | 0.07 (0.07) | 0.09 (0.07) | 43.65 (75.49) | -12.81 (73.38) | 0.12 (0.14) | 0.16 (0.16) |
| TRI | 12.56 (7.50) | 14.18 (8.54) | 8.71 (5.11) | 10.35 (8.04) | **8.33 (48.15)*** | **16.93 (48.10)*** | 11.95 (14.66) | 15.65 (10.92) |
| ApEn | 0.81 (0.42) | 0.96 (0.65) | 0.61 ± 0.15 | 0.61 ± 0.15 | 27.26 (29.69) | 20.92 (36.06) | 0.89 (0.43) | 0.16 (0.16) |
| SamEn | 0.75 (0.59) | 1.11 (1.25) | 1.01 (0.84) | 1.36 (1.12) | 2.71 (58.99) | -43.52 (110.11) | **0.59 (0.64)*** | **0.97 (0.48)*** |
| SD1 | 68.09 (53.36) | 91.80 (103.46) | 65.80 (65.77) | 86.86 (89.15) | -14.98 (102.05) | -19.78 (49.17) | 122.29 (49.25) | 0.97 (1.00) |
| SD2 | 120.71 (111.16) | 153.35 (71.59) | 105.28 (104.39) | 127.50 (75.90) | -10.72 (67.00) | -2.39 (53.58) | 156.26 (130.45) | 145.35 (88.18) |
| SD1SD2ratio | 0.54 ± 0.22 | 0.60 ± 0.28 | 0.65 ± 0.32 | 0.70 ± 0.34 | -2.25 (62.79) | -25.10 (50.02) | 0.75 (0.42) | 185.67 (93.90) |
| DFA alpha 1 | 0.75 (0.22) | 0.81 (0.36) | 0.79 (0.43) | 0.87 (0.54) | -5.95 (72.51) | -20.17 (57.27) | -1.07e-15 ± 4.36e-16 | -1.09e-15 ± 5.04e-16 |
| DFA alpha 2 | 0.96 ± 0.25 | 0.91 ± 0.32 | 0.83 (0.47) | 0.89 (0.68) | 13.96 (81.63) | -23.13 (67.83) | -2.43e-16 ± 7.92e-16 | -2.03e-16 ± 7.35e-16 |

**REFERENCES**

Corino, Valentina D.A., Eros Montin, Antonella Messina, Paolo G. Casali, Alessandro Gronchi, Alfonso Marchianò, and Luca T. Mainardi. 2018. “Radiomic Analysis of Soft Tissues Sarcomas Can Distinguish Intermediate from High-Grade Lesions.” *Journal of Magnetic Resonance Imaging* 47 (3): 829–40. https://doi.org/10.1002/jmri.25791.
